# The Neuroprotective Effect of Isotetrandrine on Parkinson's Disease via Anti-Inflammation and Antiapoptosis *In Vitro* and *In Vivo*

**DOI:** 10.1155/2023/8444153

**Published:** 2023-10-10

**Authors:** Ching-Hu Wu, Kun-Ling Lin, Cheng-Yu Long, Chien-Wei Feng

**Affiliations:** ^1^Department of Obstetrics and Gynecology, Kaohsiung Medical University Hospital, Kaohsiung Medical University, Kaohsiung 80708, Taiwan; ^2^Department of Obstetrics and Gynecology, Kaohsiung Municipal Ta-Tung Hospital, Kaohsiung Medical University, Kaohsiung 80145, Taiwan; ^3^Department of Obstetrics and Gynecology, College of Medicine, Kaohsiung Medical University, Kaohsiung 80708, Taiwan; ^4^Department of Obstetrics and Gynecology, Kaohsiung Municipal Siao-Gang Hospital, Kaohsiung Medical University, Kaohsiung 81267, Taiwan; ^5^Regenerative Medicine and Cell Therapy Research Center, Kaohsiung Medical University, Kaohsiung 80708, Taiwan; ^6^Center for Cancer Research, Kaohsiung Medical University, Kaohsiung 80708, Taiwan

## Abstract

Parkinson's disease (PD) is one of the most influential diseases in the world, and the current medication only can relieve the clinical symptoms but not slow the progression of PD. Therefore, we intend to examine the neuroprotective activity of plant-derived compound isotetrandrine (ITD) *in vitro* and *in vivo. In vitro*, cells were cotreated with ITD and LPS to detect the inflammatory-related protein and mRNA. *In vivo*, zebrafish were pretreated with ITD and inhibitors prior to 6-OHDA treatment. Then, the behavior was monitored at 5 dpf. Our result showed ITD inhibited LPS-induced upregulation of iNOS, COX-2 protein expression, and iL-6, inos, cox-2, and cd11b mRNA expression in BV2 cells. The data in zebrafish also demonstrated a significant improvement of ITD on the 6-OHDA-induced locomotor deficiency. ITD also improved 6-OHDA-induced apoptosis in zebrafish PD. We also pharmacologically validated the mechanism with three inhibitors, including LY294002, PI3K inhibitor; LY32141996, ERK inhibitor, SnPP, and HO-1 inhibitors. All of these inhibitors could abolish the neuroprotective effect of ITD partially in locomotor activity. Besides, the molecular level also showed the same trend. Treatment of these inhibitors could significantly abolish ITD-induced antineuroinflammatory and antioxidative stress effects in zebrafish PD. Our study showed ITD possessed a neuroprotective activity in zebrafish PD. The mRNA level also supported our arguments. The neuroprotection of ITD might be through antineuroinflammation and antiapoptosis pathways via PI3K, ERK, and HO-1.

## 1. Introduction

Parkinson's disease (PD) is one of the most widespread neurodegenerative diseases in the world [1]. The patients typically exhibit some typical symptoms including tremors, stiffness in the limbs and joints, slow movements, reduced coordination, and slow swallowing function [2]. In clinical, the first-line treatment for PD is still medication including levodopa, dopamine agonists, monoamine oxidase type B, or catechol-o-methyl-transferase inhibitor [3–5]. However, some adverse effects occurred after the administration of these drugs such as vomiting, nausea, and low blood pressure [6, 7]. Besides, the administration of medication only can relive the symptoms but not slow the progression of PD [8]. Therefore, there is an urgent demand on novel PD drugs. Among all the sources of novel compounds, the natural products derived from plants occupied a niche in recent years.

Nowadays, a rise of plant-derived therapies or nutritional supplements occurred under the coronavirus apex in a few years [9]. There were more than 30 plants in extracts or compounds, such as *Artemisia annua*, *Houttuynia cordata Thunb*, and *Sambucus formosana Nakai*, which possessed antiviral activity via their own mechanism during the viral infection, respectively [10]. In PD, more kinds of preclinical compounds were discovered in plant-based database. There were about 56 formulations or molecules which possessed the neuroprotective activity [11]. Most of them protect dopaminergic cell against neurotoxin to reduce the risk of degeneration [12, 13]. Hence, our study also intended to verify a plant-based compound on its neuroprotective activity.

Isotetrandrine (ITD) is a botanic natural product that originally came from *Berberis bealei* and *Berberis duclouxiana* [14]. It is considered as an analgesic, antimicrobial, immunosuppressive, and antimalarial agent [15]. It is also known as a cell-permeable PLA2 inhibitor and has also shown to inhibit *α*1 adrenoceptor [16, 17]. However, the neuroprotective activity of ITD has not been investigated yet. Our study intended to realize the neuroprotective effect of ITD and clarified its mechanism of action via various inhibitors. We hoped the data and subsequent research could provide a new choice for the suffering PD patients.

## 2. Materials and Methods

### 2.1. Reagents

Isotetrandrine was purchased from Enzo Life Sciences, Inc. (Enzo Life Sciences, USA, NY, No, 477-57-6), which is also called Berbamine methyl ether, 6,6′,7,12-Tetramethoxy-2,2′-dimethyl-berbaman in IUPAC. LY294002, PI3K inhibitor (Selleck Chemicals LLC, USA, TX, No. S1105) and LY3214996, ERK1/2 inhibitor (Selleck Chemicals LLC, USA, TX, No. S8534) were purchased from Selleckchem Chemicals and SnPP (MedChemExpress LLC, USA, NJ, No. HY-101194) was purchased from MedChemExpress LLC.

### 2.2. Cell Maintain

BV2 murine microglia cell line was purchased from Elabscience Biotechnology Inc (Elabscience, USA, TX, No. CRL-2266). The cells were maintained with BV2 cells complete medium (Elabscience, USA, TX, No. CM-0493) in a 5% CO_2_ 37°C incubator. The cells were cotreated with different concentrations (10, 100, and 200 *μ*M) of ITD and LPS (1 *μ*g/ml) for 24 hr and harvested the cells for the western blot and qPCR analysis. The induction condition was referred to in a previous study [18]. The cell number used for western blot or qPCR was 1 × 10^6^ per 6 cm dish.

### 2.3. Quantitative PCR Analysis in Cell or Zebrafish

In the cell, the cells were cotreated with different concentrations (10, 100, and 200 *μ*M) of ITD for 24 hr, and LPS (1 *μ*g/ml) was harvested cells for testing. In zebrafish, ITD (200 *μ*M) with or without LY294002 (20 *μ*M), LY3214996 (20 *μ*M), and SnPP (40 *μ*M) was applied to zebrafish embryos for 87 hours (9 hours postfertilization (hpf) to 5 days postfertilization (dpf)) in the presence of 6-OHDA (250 *μ*M) (2–5 dpf). At 5 dpf, the qPCR and protein samples were collected, respectively. The TRIzol reagent was used to extract total RNA from 20 zebrafish larvae in each treatment group in accordance with the manufacturer's instructions (Fisher Scientific, USA, MA, No. 15596026). Using the iScript cDNA synthesis kit, the reverse transcription of RNA into single-stranded cDNA was performed. Then, we used the Bio-Rad real-time PCR apparatus to conduct real-time PCR using the iQTM SYBR_Green supermix (Bio-Rad, USA, CA, No. 172–5270). Using the GAPDH as the internal reference, the relative fold change (log2 ratio), each gene's level were determined. The primers we used were referred from previous studies [19–22] and listed as follows: *cd11b*: F-5′-CAGATCAACAATGT-GACCGTATGGG-3′, R-5′-CATCATGTCCTTGTACTGC-CGCTTG-3′; *Il-6*: F-5′-CCACTTCACAAGTCGGAGGC-3′, R-5′-CCAGCTTATCTGTTAGGAGA-3′; F-*Inos*: 5′-ATGTGGTACTCAGCGTGCTCCAC-3′, R-5′-CCACAATAGTACAATACTACTTGG-3′; *Cox-2*: F-5′-GAACATTGTGAACAACATCCCC-3′, R-5′-GGTGGCATACATCATCAGACC-3′. *Gapdh*: F-5′-TTGCAGTGGCAAAGTGGAGA-3′, R-5′-CGTGGTTCACACCCATCACAA-3′ in BV2 cell. *th1*: F-5′-GACGGAAGATGATCGGAGACA-3′, R-5′- CCGCCATGTTCCGATTTCT-3′;*bcl-2*: F-5′-AGGAAAATGGAGGTTGGGATG-3′, R-5′-TGTTAGGTATGAAAACG-3′; *bax*: F-5′- GGCTATTTCAACCAGGGTTCC-3′, R-5′-TGCGAATCACCAATGCTGT-3′; *ho-1*: F-5′-ATGCCCTTGTTTCCAGTCAGC-3′, R-5′-GGACTTGGAGCACTTCTTCGG-3′; *gapdh*: F-5′-CTGGGATGATGTTCTGACTGG-3′, R-5′-AGTTGTAAGCAATGCCTCCTG-3′ in zebrafish.

### 2.4. Western Blotting

In accordance with the treatment plan, zebrafish and cells were treated in 6 cm dishes. Cell pellets were collected, and an equivalent amount of lysis solution (containing 0.5 mM and 0.1% protease inhibitor cocktail) was used to lyse them. Using the Bradford protein assay, the lysed protein was then adjusted to the same concentration before being added to the sample buffer, which consists of 2% sodium dodecyl sulfate, 10% glycerol, 0.1% bromophenol blue, 2% 2-mercaptoethanol, and 50 mM Tris-HCl in pH = 7.2. SDS-PAGE electrophoretic separation was used to separate the prepared protein samples. Then, target proteins were moved to a PVDF membrane that had been activated. PVDF membranes were treated with anti-iNOS (Cayman Chemical, USA, MI, No. 160862), anti-COX-2 (Cayman Chemical, USA, MI, No. 160106), and anti-*β*-actin for an overnight period at 4°C after being blocked with 5% nonfat dried milk. Chemiluminescence was used to find a secondary antibody coupled to horseradish peroxidase (Millipore Corp). The UVP BioChemi Imaging System was utilized to capture images, and LabWorks 4.0 software was used to calculate the relative densitometry.

### 2.5. Zebrafish Maintenance

In this work, we employed wild-type zebrafish (AB strain) and maintained in the KMU Zebrafish Core Facility at 28.5°C under a 10 h dark/14 h light night/day cycle. The embryos were acquired by natural spawning, and the normal stages were screened using conventional criteria [23]. The embryos were incubated in Hank's buffer (NaCl (13.7 mM), KCl (540 *μ*M), Na_2_HPO_4_ (25 *μ*M), KH_2_PO_4_ (44 *μ*M), CaCl_2_ (300 *μ*M), MgSO_4_ (100 *μ*M), and NaHCO_3_ (420 *μ*M).

### 2.6. Zebrafish PD Assay

ITD (200 *μ*M) with or without LY294002 (20 *μ*M), LY3214996 (20 *μ*M), and SnPP (40 *μ*M) were applied to zebrafish embryos for 87 hours (9 hpf to 5 dpf) in 24-well microplate. From 2 to 5 dpf, we cotreated with 6-OHDA (250 *μ*M). At 5 dpf, the qPCR and protein samples were collected, respectively. The zebrafish behavioral tests alluded to in a prior work at 5 dpf and 6 dpf [24]. We performed the behavior tests as previous study [25]. In brief, zebrafish larvae were placed in the cuvette, and their behavior was tracked using an automated video tracking system (Singa Technology Co.; Taiwan, No, TM-01). The cuvette utilized in this investigation was 1-1-4.5 cm (L-W-H) in size and was held in front of the camera for 7.5 cm. Each zebrafish in the test was given a 2-minute adaption period before the swimming pattern and the total distance of each fish was recorded for 5 minutes.

### 2.7. Statistical Analysis

Results were presented as mean ± SEM. For western blotting data, the intensity of each band was expressed as the relative OD divided by the average OD values from all internal controls. Data were analyzed using one-way analysis of variance followed by Dunnett's test. The *p* values less than 0.05 were considered statistically significant.

## 3. Results

### 3.1. The Anti-Inflammatory Effect of ITD on LPS-Induced Inflammation in BV2 Cell

**Figure 1:**
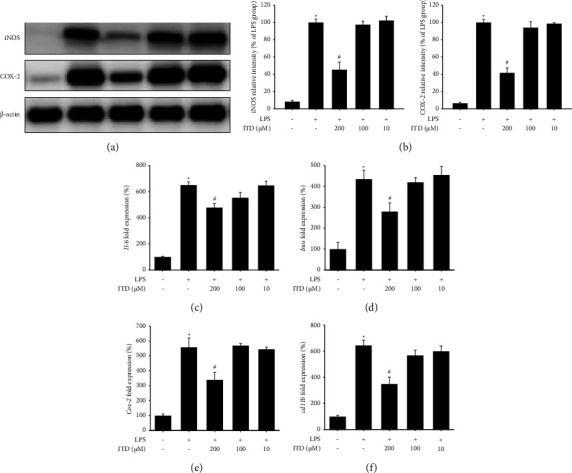
Effect of isotetrandrine (ITD) on LPS-induced iL-6, iNOS, and COX-2 mRNA and protein expression in BV2 cell. BV2 microglia cells were cotreated with LPS and different concentrations of isotetrandrine (ITD) (200, 100, and 10 *μ*M) for 24 hr and harvested for western blots and qPCR. (a) Western blots for iNOS, COX-2, and *β*-actin protein expression in each group. (b) Quantification results of relative density of iNOS and COX-2 expression in each group. The relative density of the LPS-induced group was taken to be 100%. *β*-actin was used as an internal control. (c–f) Quantitative PCR of interleukin-6 (*Il-6*), inducible nitric oxide synthase (*Inos*), and cyclooxygenase-2 (*Cox-2*) and *cd11b* mRNA expression in each group. *Gapdh* was used as an internal control. Data are presented as mean ± SEM (*n* = 3). ^*∗*^Significantly different from the control group; *p*  <  0.05; ^#^significantly different from the LPS group.

We assessed the antineuroinflammation effect of ITD on LPS-induced BV2 cell in western blots. ITD (10, 100, or 200 *μ*M) was cotreated with LPS (1 *μ*g/ml) for 24 hr and harvested for the following western blots. Our data showed that 200 *μ*M ITD significantly inhibited LPS-induced upregulation of inducible nitric oxide synthase (iNOS) and cyclooxygenase-2 (COX-2) protein expression (Figure 1(a)). The original blots are shown in supplemental figure 1 (Figure S1). The quantification results of relative density also showed only 200 *μ*M ITD apparently ameliorated LPS-induced upregulation of iNOS and COX-2, and 100, 10 *μ*M ITD did not affect the expression (Figure 1(b)). Then, we evaluated the interleukin-6 (*Il-6*), *Inos,* and *Cox-2* mRNA levels to confirm the finding. ITD (10, 100, or 200 *μ*M) was cotreated with LPS (1 *μ*g/ml) for 24 hr and harvested for qPCR. Our data demonstrated that 200 *μ*M ITD could significantly reduce LPS-induced upregulation of *Il-6* (Figure 1(c)), *Inos* (Figure 1(d)), *Cox-2* (Figure 1(e)), and *cd11b* (Figure 1(f)) mRNA expression.

### 3.2. The Protective Effect of ITD on 6-OHDA-Induced Locomotor Deficiency in Zebrafish

**Figure 2:**
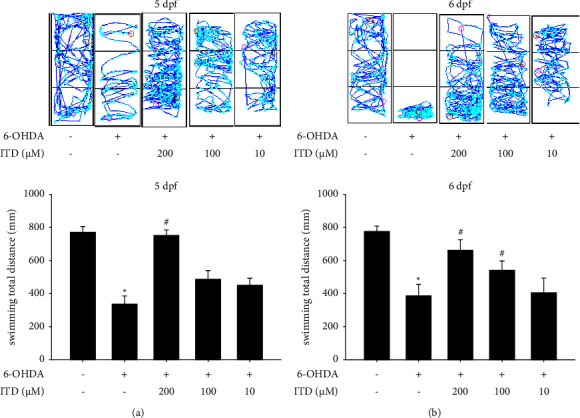
Effect of ITD on 6-OHDA-induced locomotor deficiency in zebrafish. Zebrafish larvae were pretreated with 200, 100, or 10 *μ*M ITD from 9 h postfertilization (hpf) to 5 dpf and then challenged with 250 *μ*M 6-OHDA from 2 to 5 dpf. The locomotor activity of zebrafish was measured at (a) 5 dpf and (b) 6 dpf. The upper panel shows a representative pattern, and the lower panel shows average results. Data are presented as mean ± SEM (*n* = 16). ^*∗*^significantly different from the control group; ^#^significantly different from the 6-OHDA group; *p*  <  0.05.

A Zebrafish PD assay was performed to assess the neuroprotective effect of ITD. Our results showed that exposure to 250 *μ*Μ 6-OHDA (2–5 dpf) reduced the total swimming distance from 775.1 ± 35.3 to 341.6 ± 49.0 mm. However, pretreatment with 200 *μ*Μ ITD (9 hpf to 5 dpf) significantly reversed the total swimming distance from 341.6 ± 49.0 to 756.3 ± 33.9 mm at 5 dpf (Figure 2(a)). However, 10 and 100 *μ*Μ ITD did not show an obvious impact on the zebrafish locomotor deficiency. Moreover, the data also showed that 6-OHDA treatment significantly decreased the total swimming distance from 780.9 ± 31.8 to 391.5 ± 69.3 at 6 dpf (Figure 2(b)). The pretreatment of 200 *μ*Μ and 100 *μ*Μ ITD significantly reversed 6-OHDA-induced down-regulation of total swimming distance (Figure 2(b)).

### 3.3. The Anti-Inflammatory Effect of ITD on 6-OHDA-Induced Modulation in Zebrafish PD

**Figure 3:**
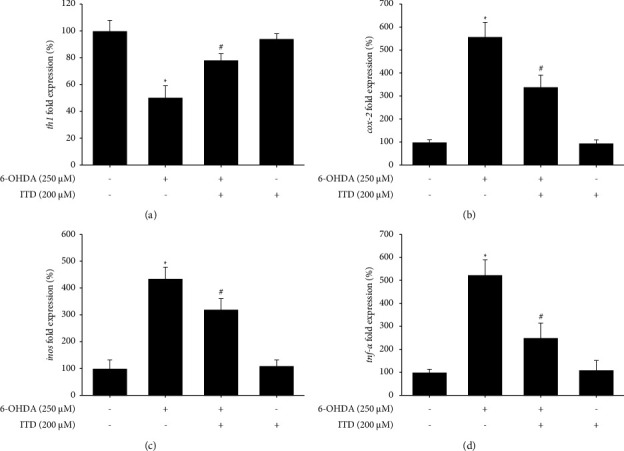
Effect of ITD on 6-OHDA-induced modulation of inflammation-related mRNA expression in zebrafish. Zebrafish larvae were pretreated with 200 *μ*M ITD from 9 hpf to 5 dpf and then challenged with 250 *μ*M 6-OHDA from 2 to 5 dpf and harvested at 5 dpf for qPCR. (a–d) Quantitative PCR results of tyrosine hydroxylase (*th1*), *inos*, *cox-2,* and tumor necrosis factor-*α* (*tnf-α*) in each group. Data are presented as mean ± SEM. Each sample contained pooled material from twenty fish. ^*∗*^significantly different from the control group; ^#^significantly different from the 6-OHDA group, *p*  <  0.05.

We then examined the mRNA level of inflammation-related genes such as inducible nitric oxide synthase (iNOS), cyclooxygenase-2 (COX-2), and tumor necrosis factor-alpha (TNF-*α*). Besides, we also examined tyrosine hydroxylase (TH), the rate-limiting enzyme of catecholamine synthesis, to assess the number of dopamine neurons encoded by *th1*. Our data showed that 6-OHDA treatment could significantly reduce *th1* expression, and pretreatment of ITD significantly reversed 6-OHDA-induced down-regulation of *th1* expression (Figure 3(a)). The inflammation-related gene also showed the same trend. The 6-OHDA treatment showed a significant increase inos, cox-2, and tnf-*α* mRNA expression. Moreover, the pretreatment of 200 *μ*M ITD significantly ameliorated 6-OHDA-induced upregulation of inos (Figure 3(b)), cox-2 (Figure 3(c)), and tnf-*α* (Figure 3(d)) expression.

### 3.4. Effect of PI-3K Inhibitor on Modulation of the ITD Neuroprotective Effect in 6-OHDA-Induced Zebrafish Deficiency

**Figure 4:**
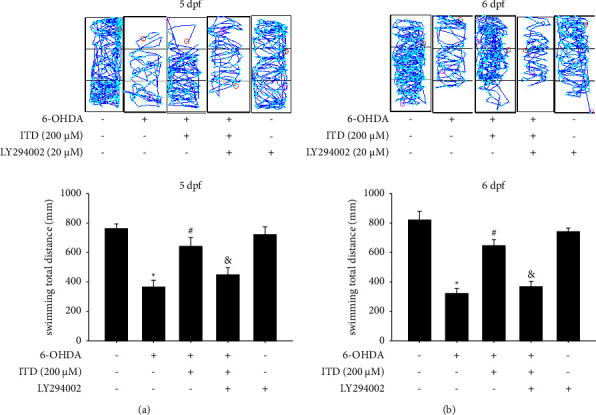
Effect of LY294002 on ITD neuroprotective effect in 6-OHDA-treated zebrafish. Zebrafish larvae were pretreated with 20 *μ*M LY294002 and 200 *μ*M ITD from 9 hpf to 5 dpf and then challenged with 250 *μ*M 6-OHDA from 2 to 5 dpf. The locomotor activity was measured at (a) 5 dpf and (b) 6 dpf. The upper panel shows a representative pattern, and the lower panel shows average results. Data are presented as mean ± SEM (*n* = 16). ^*∗*^significantly different from the control group; ^#^significantly different from the 6-OHDA group; &, significantly different from the 6-OHDA plus ITD group, *p*  <  0.05.

We then used PI3K inhibitor LY294002 to clarify ITD mechanism of action. The result depicted that the 250 *μ*M 6-OHDA treatment (2 to 5 dpf) could decrease total swimming distance from 767.6 ± 31.7 to 369.8 ± 45.5 mm. The pretreatment of 200 *μ*M ITD (9 hpf–5 dpf) could reverse 6-OHDA-induced down-regulation of total swimming distance at 5dpf from 369.8 ± 45.5 to 645.3 ± 61.8 mm. However, the cotreatment 20 *μ*M LY294002 (9 hpf–5 dpf) could significantly abolish the neuroprotective effect of ITD from 645.3 ± 61.8 to 452.1 ± 49.5 mm at 5 dpf (Figure 4(a)). The data at 6 dpf also showed the same trend. The cotreatment 20 *μ*M LY294002 (9 hpf–5 dpf) could significantly abolish the neuroprotective effect of ITD from 650.4 ± 42.4 to 372.8 ± 36.6 mm (Figure 4(b)). The LY294002 treatment alone did not affect the locomotor activity of zebrafish in both 5 dpf and 6 dpf.

### 3.5. Effect of ERK Inhibitor on Modulation of the ITD Neuroprotective Effect in 6-OHDA-Induced Zebrafish Deficiency

**Figure 5:**
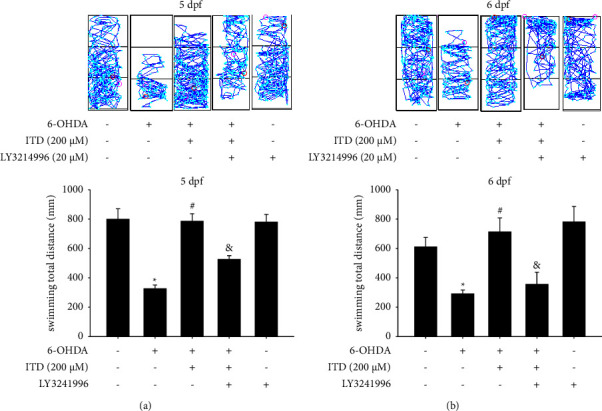
Effect of LY3214996 on ITD neuroprotective effect in 6-OHDA-treated zebrafish. Zebrafish larvae were pretreated with 20 *μ*M LY3214996 and 200 *μ*M ITD from 9 hpf to 5 dpf and then challenged with 250 *μ*M 6-OHDA from 2 to 5 dpf. The locomotor activity was measured at (a) 5 dpf and (b) 6 dpf. The upper panel shows a representative pattern, and the lower panel shows average results. Data are presented as mean ± SEM (*n* = 16). ^*∗*^significantly different from the control group; ^#^significantly different from the 6-OHDA group; &, significantly different from the 6-OHDA plus ITD group; *p*  <  0.05.

ERK1/2 inhibitor LY3214996 was also used to verify ITD mechanism of action. The data demonstrated that the 250 *μ*M 6-OHDA treatment (2 to 5 dpf) could decrease total swimming distance from 798.8 ± 72.0 to 326.2 ± 25.1 mm. The pretreatment of 200 *μ*M ITD (9 hpf–5 dpf) could reverse 6-OHDA-induced down-regulation of total swimming distance at 5dpf from 326.2 ± 25.1 to 785.1 ± 51.5 mm. However, the cotreatment 20 *μ*M LY3214996 (9 hpf to 5 dpf) could significantly abolish the neuroprotective effect of ITD from 785.1 ± 51.5 to 526.2 ± 25.1 mm at 5 dpf (Figure 5(a)). The data at 6 dpf also showed the same trend. The cotreatment 20 *μ*M LY294002 (9 hpf–5 dpf) could significantly abolish the neuroprotective effect of ITD from 650.4 ± 42.4 to 372.8 ± 36.6 mm (Figure 5(b)). The LY3214996 treatment alone did not affect the locomotor activity of zebrafish in both 5 dpf and 6 dpf.

### 3.6. Effect of HO-1 Inhibitor on Modulation of the ITD Neuroprotective Effect in 6-OHDA-Induced Zebrafish Deficiency

**Figure 6:**
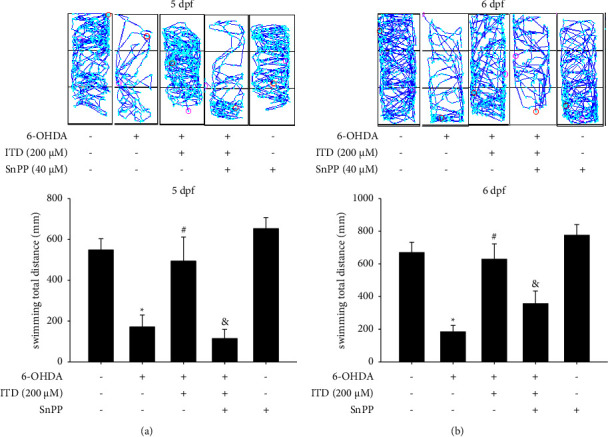
Effect of SnPP on ITD neuroprotective effect in 6-OHDA-treated zebrafish. Zebrafish larvae were pretreated with 40 *μ*M SnPP and 200 *μ*M ITD from 9 hpf to 5 dpf and then challenged with 250 *μ*M 6-OHDA from 2 to 5 dpf. The locomotor activity was measured at (a) 5 dpf and (b) 6 dpf. The upper panel shows a representative pattern, and the lower panel shows average results. Data are presented as mean ± SEM (*n* = 16). ^*∗*^significantly different from the control group; ^#^significantly different from the 6-OHDA group; &, significantly different from the 6-OHDA plus ITD group; *p*  <  0.05.

The previous study demonstrated that ITD could enhance HO-1 expression [26]. Therefore, HO-1 inhibitor, SnPP was also used to verify ITD mechanism of action. The data demonstrated that the 250 *μ*M 6-OHDA treatment (2 to 5 dpf) could decrease total swimming distance from 551.7 ± 55.7 to 175.1 ± 58.5 mm. The pretreatment of 200 *μ*M ITD (9 hpf–5 dpf) could reverse 6-OHDA-induced down-regulation of total swimming distance at 5dpf from 175.1 ± 58.5 to 497.3 ± 118.2 mm. However, the cotreatment 40 *μ*M SnPP (9 hpf–5 dpf) could significantly abolish the neuroprotective effect of ITD from 497.3 ± 118.2 to 117.9 ± 145.2 mm at 5 dpf (Figure 6(a)). The data at 6 dpf also showed the same trend. The cotreatment 40 *μ*M SnPP (9 hpf–5 dpf) could significantly abolish the neuroprotective effect of ITD from 633.4 ± 93.8 to 361.8 ± 77.0 mm (Figure 6(b)). The SnPP treatment alone did not affect the locomotor activity of zebrafish in both 5 dpf and 6 dpf.

### 3.7. Effect of PI3K, ERK, and HO-1 Inhibitors on Modulation of the ITD Neuroprotection-Related mRNA Level in 6-OHDA-Induced Zebrafish Deficiency

**Figure 7:**
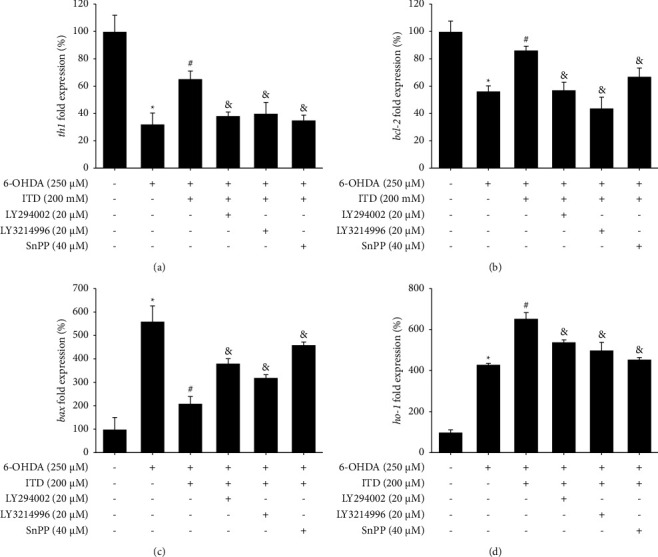
Effect of LY294002, LY3214996, and SnPP on ITD antiapoptotic effect in 6-OHDA-treated zebrafish in mRNA level. Zebrafish larvae were pretreated with 20 *μ*M LY294002, 20 *μ*M LY3214996, or 40 *μ*M SnPP, and 200 *μ*M ITD from 9 hpf to 5 dpf and then challenged with 250 *μ*M 6-OHDA from 2 to 5 dpf and harvested at 5 dpf for qPCR. Quantitative PCR results of (a) *th1*, (b) *bcl-2*, (c) *bax,* and (d) *ho-1* at (a) 5 dpf and (b) 6 dpf. Data are presented as mean ± SEM (*n* = 3). Each sample contained pooled material from twenty fish. ^*∗*^significantly different from the control group; ^#^significantly different from the 6-OHDA group; &, significantly different from the 6-OHDA plus ITD group; *p*  <  0.05.

We then evaluated some apoptotic genes such as *th1*, *bcl-2*, *bax,* and oxidative-related gene *ho-1* in the absence of the inhibitors. Our result demonstrated that 250 *μ*M 6-OHDA (2 to 5 dpf) treatment significantly decreased *th1* and *bcl-2* mRNA levels and increased *bax* and *ho-1* mRNA levels. Then, the pretreatment of 200 *μ*M ITD (9 hpf–5 dpf) significantly reversed 6-OHDA-induced down-regulation of *th1* and *bcl-2* and inhibited 6-OHDA-induced upregulation of *bax* and *ho-1* mRNA level. However, the cotreatment of LY294002 (20 *μ*M), LY3214996 (20 *μ*M), and SnPP (40 *μ*M) significantly abolished the recovery of *th1* caused by ITD (Figure 7(a)). Further, the cotreatment of LY294002 (20 *μ*M), LY3214996 (20 *μ*M), and SnPP (40 *μ*M) significantly abolished the modulation of apoptotic gene *bcl-2* and *bax* caused by ITD (Figures 7(b) and 7(c)). Finally, the enhancement of ITD on ho-1 mRNA level was all eliminated by the cotreatment of LY294002 (20 *μ*M), LY3214996 (20 *μ*M), and SnPP (40 *μ*M) (Figure 7(d)).

## 4. Discussion

In our study, we demonstrated the antineuroinflammation activity of ITD in BV2 murine microglia with iNOS, COX-2, and iL-6 mRNA and protein levels. We further confirmed the neuroprotective activity of ITD in the zebrafish PD model. Our data also showed a significant improvement of ITD on 6-OHDA-induced locomotor deficiency in zebrafish. The qPCR analysis of apoptotic-related mRNA expression also demonstrated that ITD could improve 6-OHDA-induced apoptosis in the zebrafish PD model. Besides, we also pharmacologically validated the mechanism with three inhibitors, included LY294002, PI3K inhibitor; LY32141996, ERK inhibitor; and SnPP, HO-1 inhibitor. All of these inhibitors could abolish the neuroprotective effect of ITD partially in locomotor activity. The molecular level also showed the same trend. Treatment of these inhibitors could significantly abolish ITD-induced antiinflammatory and antioxidative stress effects in zebrafish PD assay. Other antiinflammatory compounds were also discussed about their neuroprotection activity.

Previous studies used BV2 murine microglia as an *in vitro* model to investigate the mechanism of neuroinflammation since 1990 [27]. It was evaluated by microglia biomarkers and tested 90% positive for nonspecific esterase activity. Besides, BV2 didn't show the signal of glial fibrillary acidic protein (GFAP) or galactocerebroside (GC) for astrocyte and oligodendrocytes, respectively, which is similar to primary microglia [28]. Hyperactivated microglia serves as an essential function in regulating neuroinflammatory responses that induce neuronal injury. In recent years, there has been a lot of focus on finding innovative ways to treat neuroinflammation [29, 30]. Velagapudi et al. demonstrated that tiliroside not only inhibited BV2 microglia activation by LPS/IFN*γ*-induced neuroinflammation but also protected HT22 neuronal against damage via targeting Nrf2 antioxidative pathways. The compound could inhibit NF-*κ*B acetylation by activating SIRT1. In *in vivo* model, tiliroside also enhanced SIRT1 activity in mice hippocampus neurons [31]. Besides, Park et al. demonstrated that Gyejibokryeong-hwan (GBH), a traditional Korean medicine, could ameliorate LPS-stimulated BV2 activation in NO production, inflammatory cytokines and reduced iNOS, COX-2, TNF-*α*, and IL-6 protein expression. In addition, inflammatory transcription factor NF-) *κ*B p65 was also decreased under the GBH treatment. GBH could also enhance nuclear factor-E2-related factor 2/cAMP response element-binding protein-dependent antioxidant pathway and the downstream HO-1 protein expression [21]. Our data also revealed that 200 *μ*M ITD could significantly inhibit LPS-induced upregulation of iNOS and COX-2 in mRNA and protein levels (Figure 1). Except for the *in vitro* model, some studies also used zebrafish as a screening tool in antiPD drugs.

Bretaud et al. first demonstrated that zebrafish could be used as a PD animal model [32]. They treated neurotoxins in zebrafish larvae and intended to induce PD-like symptoms, including reducing swimming total distance or swimming velocity. Besides, Anichtchik et al. also showed that intracerebral injections of 6-OHDA and MPTP lead to a significant locomotor deficit in adult zebrafish [33]. The studies mentioned above both tried to use zebrafish to imitate clinical symptoms of PD, and they also confirmed the feasibility of zebrafish as an appropriate model animal of PD. Li et al. discovered that utilizing *Sanghuangprous vaninii* methanol extracts (SvMEs) in the zebrafish repaired dopaminergic neuron loss and neurovascular decrease in a concentration-independent way. It also improved locomotor function in the MPTP-induced zebrafish PD model. In addition, SvMEs revealed a significant antioxidant activity *in vitro*, which was also demonstrated *in vivo* on ktr4:NTR-hKikGR zebrafish and they also discovered that SvMEs may reduce oxidative stress and *α*-synuclein aggregation which could alleviate PD-like symptoms. Antioxidant-related genes (*sod1*, *gss*, *gpx4a*, *gclm*, and *cat*) suggested that SvMEs possessed an anti-PD effect via an antioxidation mechanism [34]. Our previous research in 2016 demonstrated how 11-dehydrosinulariolide (11-de) exerts its therapeutic effect via triggering cytosolic or mitochondrial DJ-1 expression, which subsequently activates the downstream Akt/PI3K, p-CREB, and Nrf2/HO-1 pathways. In addition, we discovered that 11-de might rescue the 6-OHDA-induced decrease in total swimming distance in a PD zebrafish model. Utilizing a rat PD model, we demonstrated that an increase in the number of rotations and time spent on the beam caused by 6-OHDA could be reversed by 11-de treatment [35]. Kozioł et al. demonstrated that the 7.5 *μ*M xanthotoxin treatment restored locomotor activity deficiency in zebrafish larvae. Meanwhile, administration of xanthotoxin also reduced mobility deficits in a mouse model at a dose of 25 mg/kg. The same action of the same chemical in two distinct animal models implies compatibility and strengthens the feasibility of zebrafish-based *in vivo* models. Their findings also suggested that xanthotoxin might be a promising lead compound [36]. Our current finding also depicted that ITD could significantly reverse 6-OHDA-induced locomotor deficit (Figure 2) and reflected on the molecular level in apoptotic and inflammation mRNA level (Figure 3).

Indeed, ITD was mentioned as an antiinflammatory agent in previous studies. Liang et al. showed that the cytokine levels of TNF-*α*, IL-1*β*, and IL-6 in supernatant were reduced by ITD. Furthermore, their result showed that ITD significantly inhibited the activation of MAPK and NF-*κ*B, which are induced by LPS in acute lung injury (ALI) model. Their findings showed that ITD reduced the severity of LPS-induced ALI via inactivating MAPK and NF-*κ*B. ITD might inhibit oxidative stress and the pulmonary inflammatory process [37]. Sun et al. demonstrated that 100 *μ*M ITD could ameliorated astrocyte cytotoxicity in neuromyelitis optica via inhibiting the binding of neuromyelitis optica-immunoglobulin G to aquaporin 4 [38]. Moreover, ITD reduced tert-butyl hydrogen peroxide-induced oxidative damage via increasing HO-1 protein expression. This was accomplished by separating the nuclear translocation of factor-erythroid 2 p45-related factor 2 (Nrf2) from the Nrf2-Keap1 complex via activation of extracellular signal-regulated protein kinase and c-Jun NH2-terminal kinase, as well as inactivation of Keap1 [26]. In their study, they also used five inhibitors, including SB203580 (p38 inhibitor), LY294002 (PI3K/Akt inhibitor), SP600125 (JNK inhibitor), U0126 (ERK inhibitor), or SnPP (HO-1 inhibitor) to further clarify the mechanism of ITD. To our surprise, the protective effect of ITD was abolished by the pretreatment of SnPP but not in LY294002. Our data showed that the neuroprotective effect of ITD was significantly inhibited by the treatment of SnPP, LY294002, and LY3214996 (Figures 4–6). We suggested that the integrality of *in vivo* model (zebrafish) leads to differences in results.

**Figure 8:**
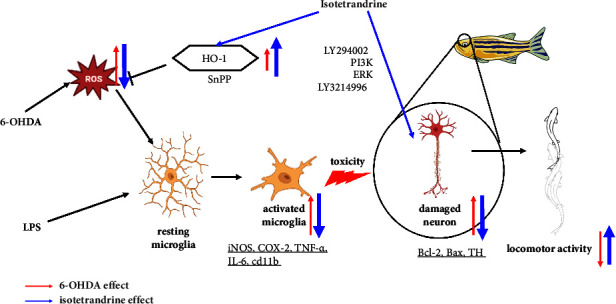
Schematic diagram of mechanism of ITD in 6‐OHDA‐induced cell death.

In conclusion, our results demonstrated that ITD possessed an antineuroinflammatory activity in LPS-induced BV2 murine microglia cell. Furthermore, it also showed a great neuroprotective activity in zebrafish locomotor deficit. The mRNA level also supported our arguments. We also verified the mechanism of action via PI3K, ERK, and HO-1 inhibitors and proved ITD's effect on antineuroinflammation and antiapoptosis pathway. We believed ITD may be a promising candidate for PD therapy (Figure 8).

## 5. Conclusions

6-OHDA treatment significantly increases intracellular ROS, which activates the resting microglia. The inflammatory cytokine such as TNF-*α* and iL-6 leads to dopaminergic cell death in zebrafish. The reduction of dopamine neurons is also reflected in locomotor deficiency. The pretreatment of ITD could protect cells from 6-OHDA damage in two ways. First, ITD inhibited LPS or 6-OHDA-induced upregulation of iNOS, COX-2, iL-6, cd11b, and TNF-*α* expression. In addition, ITD also protects neurons in the antiapoptosis pathway, including PI3K and p-ERK pathways. We also verified that ITD could inhibit intracellular ROS through the upregulation of HO-1 expression. Through the antiapoptotic and antineuroinflammation effect of ITD shown in BV2 cells and zebrafish, ITD could protect cells from damage and rescue zebrafish from the locomotor deficiency induced by exposure to 6-OHDA treatment.

## Data Availability

No data were used to support the findings of this study.

## Supplementary Materials

Supplementary Materials Supplemental Figure 1: Uncropped western blot image for Figure 1. The uncropped western blot of iNOS, COX-2, and *β*-actin in Figure 1 is shown. Five groups were marked as follows: C (Control), L (LPS), L + 200 (LPS + 200 *μ*M ITD), L + 100 (LPS + 100 *μ*M ITD), and L + 10 (LPS+10 *μ*M ITD).
