# Supplementary material for: The Neuroprotective Effect of Isotetrandrine on Parkinson's Disease via Anti-Inflammation and Antiapoptosis In Vitro and In Vivo
Source: Parkinsons Dis. 2023 Oct 10;2023:8444153. doi: 10.1155/2023/8444153 (PMC10581844; doi:10.1155/2023/8444153)
Supplement: Supplementary Materials — Supplemental Figure 1: Uncropped western blot image for Figure 1. The uncropped western blot of iNOS, COX-2, and β-actin in Figure 1 is shown. Five groups were marked as follows: C (Control), L (LPS), L + 200 (LPS + 200 μM ITD), L + 100 (LPS + 100 μM ITD), and L + 10 (LPS+10 μM ITD). [file 8444153.f1.docx]

**
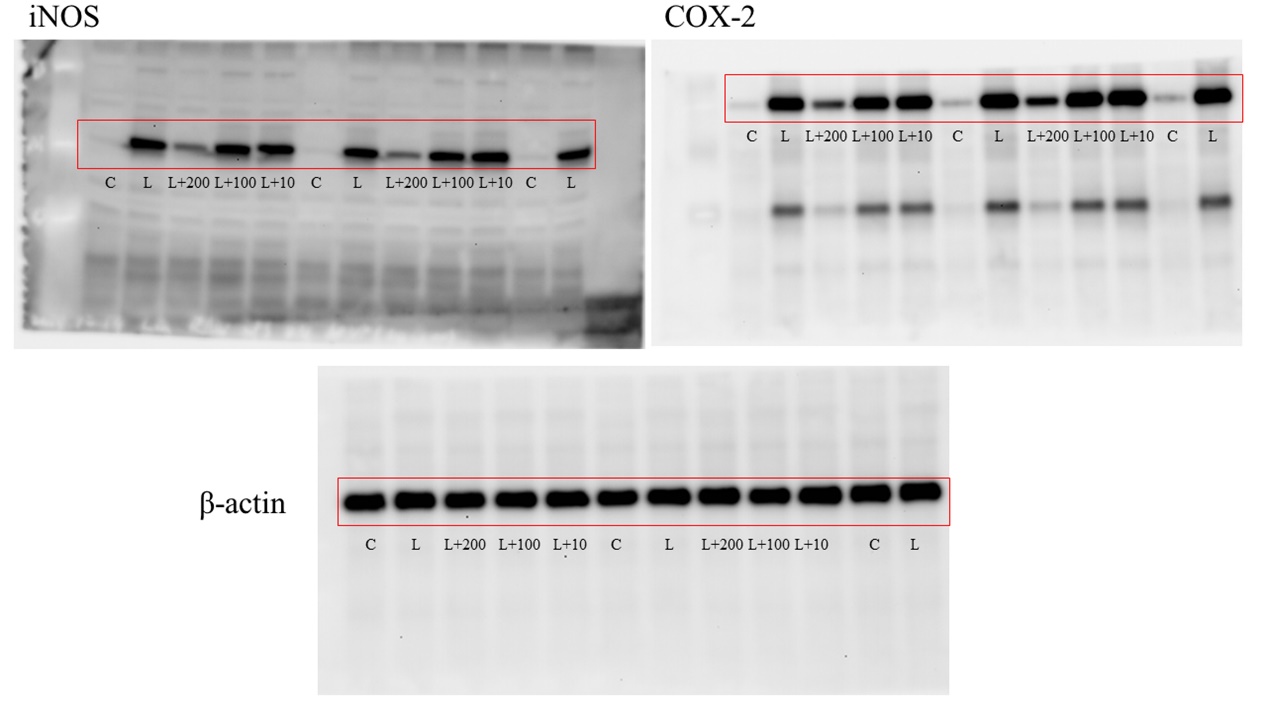
Supplementary Description:**

Supplemental Figure 1. Uncropped western blot image for Figure 1. The uncropped western blot of iNOS, COX-2 and β-actin in Figure 1 were shown. Four groups were marked as followed: C (Control), L (LPS), L+200 (LPS+200μM ITD), L+100 (LPS+100μM ITD), and L+10 (LPS+10μM ITD).
